# Prenatal air pollution exposure and the risk of transient tachypnea of the newborn

**DOI:** 10.3389/fped.2026.1941575

**Published:** 2026-10-15

**Authors:** Fang-Ju Lin, Ching-Heng Lin, Yi-Hsuan Lin, Ming-Chih Lin

**Affiliations:** 1 Children's Medical Center, Taichung Veterans General Hospital, Taichung, Taiwan; 2 Department of Medical Research, Taichung Veterans General Hospital, Taichung, Taiwan; 3 Institute of Public Health and Community Medicine Research Center, National Yang Ming Chiao Tung University, Taipei, Taiwan; 4 Department of Epidemiology and Public Health, UCL, London, United Kingdom; 5 Department of Post-Baccalaureate Medicine, College of Medicine, National Chung Hsing University, Taichung, Taiwan; 6 Institute of Public Health, National Yang Ming Chiao Tung University, Taipei, Taiwan; 7 School of Medicine, Chung Shan Medical University, Taichung, Taiwan

**Keywords:** air pollution, carbon monoxide (CO), nitrogen oxides (NOx), particulate matter (PM_2.5_), prenatal exposure, transient tachypnea of the newborn

## Abstract

**Objective:**

Although prenatal exposure to ambient air pollution is known to compromise neonatal health, its specific impact on transient tachypnea of the newborn (TTN), a prevalent cause of neonatal respiratory distress, has rarely been investigated on a population-based scale. This study aimed to evaluate the association between maternal exposure to certain air pollutants during pregnancy and the risk of TTN, as well as regional variations from a nationwide perspective.

**Methods:**

We conducted a nationwide, nested case-cohort study using the Taiwan National Health Insurance Research Database linked to the Maternal and Child Health Database (2004–2020). Newborns of primiparous mothers diagnosed with TTN (*n* = 3,091) were matched with healthy controls (*n* = 12,364) at a 1:4 ratio based on maternal age, neonatal sex, and birth date. Individual prenatal exposure across the 40 weeks preceding birth was estimated by mapping maternal residential postal codes to the nearest of 71 active air monitoring stations. Multiple logistic regression models were adjusted for comprehensive perinatal and clinical confounders.

**Results:**

Multivariate analysis demonstrated that elevated prenatal exposure to carbon monoxide (CO) was strongly associated with an increased risk of TTN (adjusted odds ratio [aOR]: 1.806, 95% CI: 1.312–2.487; *p* < 0.001). Significant risk elevations were also identified for nitrogen oxides (NOx) (aOR: 1.010, 95% CI: 1.005–1.015; *p* < 0.001) and fine particulate matter (PM_2.5_) (aOR: 1.001, 95% CI: 1.000–1.002; *p* = 0.023). Geographically stratified analysis revealed that these risks were highly pronounced in the traffic-dominated, urban basin of northern Taiwan (Taipei and New Taipei City).

**Conclusion:**

This study demonstrated that prenatal exposure to specific air pollutants, including CO, NOx, and PM_2.5_ is associated with an increased risk of TTN. Furthermore, our regional analysis suggests that traffic-related emissions may exert the greatest impact.

## Introduction

Transient tachypnea of the newborn (TTN), affecting 7% to 10% of newborns, is a common cause of neonatal respiratory distress typically caused by delayed clearance of fetal lung fluid (1–3). Maternal risk factors include gestational diabetes, asthma, and elective cesarean delivery. Fetal risk factors include male sex, gestational age under 39 weeks, perinatal asphyxia, and abnormal birth weight (4, 5). While TTN usually resolves within 48 h, symptoms can last up to 7 days (6, 7). It imposes a heavy burden on healthcare, causes significant parental anxiety, and results in maternal-infant separation that delays bonding (1, 2, 6–9). Acute complications of TTN include pulmonary air leak and persistent pulmonary hypertension of the newborn (PPHN) (1, 7, 10, 11). Furthermore, long-term associations with asthma, bronchiolitis, and other childhood wheezing syndromes raise significant concern (1, 2).

Currently, the World Health Organization reports that over 90% of the global population breathes unsafe air (12). Exposure to prenatal ambient air pollution is a known determinant of adverse perinatal outcomes, including preterm birth, preeclampsia, low birth weight, and increased neonatal intensive care admissions due to respiratory distress (13–21). Some evidence has suggested prenatal air pollution may increase the risk of asphyxia and respiratory distress in neonates, in addition with increased neonatal intensive care unit (NICU) admissions (22–24). However, its specific impact on TTN has seldom been approached.

Previous literature indicates that prenatal air pollution exposure may induce neonatal pulmonary morbidity. A U.S. cohort study by Seeni et al. demonstrated that maternal PM_2.5_ and CO exposures significantly increase the risks of asphyxia, respiratory distress syndrome, TTN (22). Furthermore, prenatal air pollution may contribute to increased NICU admissions. A U.S. case-crossover study linked acute peripartum exposure to PM_2.5_ and NO_2_ with a higher likelihood of NICU admission, while another U.S. metropolitan cohort found that whole-pregnancy NO_2_ exposure significantly increased this risk (23, 25). Conversely, a Canadian prospective cohort study reported no association between prenatal PM_2.5_ or NO_2_ and NICU admission risk, despite identifying strong links to severe respiratory distress requiring assisted ventilation (3). This discrepancy may reflect variations in pollutant sources, exposure levels, or institutional NICU admission criteria (24).

The aim of this study is to investigate the association between prenatal ambient air pollution exposure and the risk of TTN on a population-based scale.

## Methods

This is a nested case-cohort study. The main data source is the National Health Insurance Research Database (NHIRD), which comprises claim data derived from Taiwan's National Health Insurance (NHI) program. Launched in 1995, the NHI is a government-administered, single-payer system with mandatory enrollment that covers over 99% of the population (26, 27). Since 2015, the NHIRD has been integrated with additional health-related datasets by the Health and Welfare Data Center (HWDC) under the Ministry of Health and Welfare (MOHW). To enable mother–child linkage, the NHIRD was connected to the Taiwan Maternal and Child Health Database (MCHD), managed by the Health Promotion Administration (HPA) (28, 29). The primary data components utilized were the inpatient expenditures and ambulatory care expenditures files, with diagnoses coded using the International Classification of Diseases, Ninth and Tenth Revisions, Clinical Modification (ICD-9-CM and ICD-10-CM). To ensure data security and patient confidentiality, all analyses were conducted on-site at the HWDC via secure access to MOHW-maintained servers. The study protocol was approved by the Institutional Review Board of Taichung Veterans General Hospital (approval number: CE16270B), and the requirement for written informed consent was waived. Because siblings are highly related, we recruited only one child per family to prevent clustering effects.

### Study population

Figure 1 illustrates the study flowchart for patient enrollment. The case group comprised newborns diagnosed with TTN (identified by ICD-9-CM code 770.6 or ICD-10-CM code P22.1) born to primiparous mothers (those in their first pregnancies), while neonates with congenital anomalies were excluded. Control newborns were randomly selected and matched at a 4:1 control-to-case ratio based on maternal age, neonatal sex, and date of birth. Due to the availability of air pollutant exposure data from 2004 to 2020, this timeframe was selected as the study period. Retrieved covariates included neonatal profiles (gestational age, birth weight, and delivery mode), maternal characteristics (age, comorbidities, and pregnancy-related complications), and place of residence.

**Figure 1:**
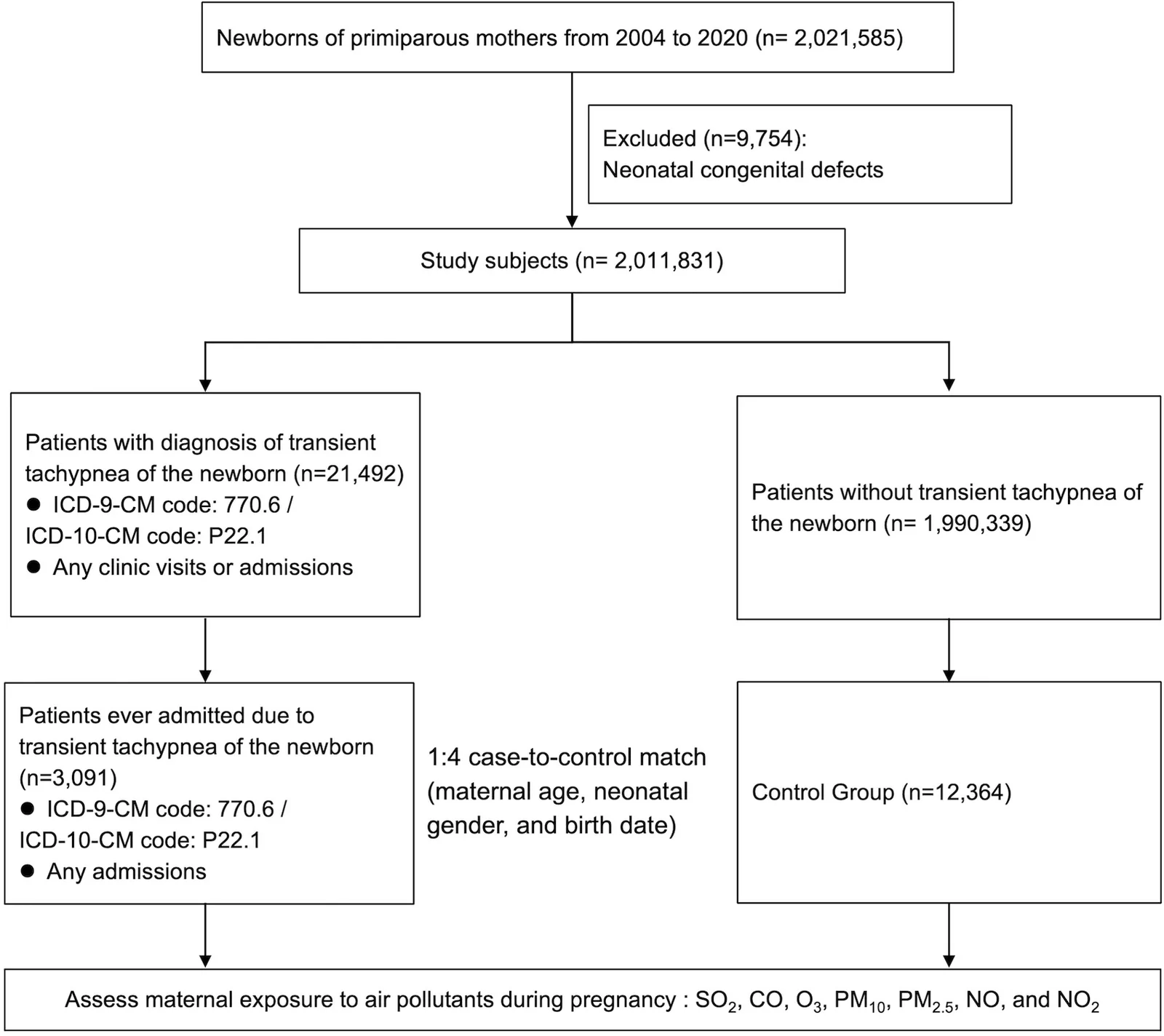
Flowchart of the study population enrollment and selection process.

### Assessment of exposure to air pollutants

The Taiwan Air Quality Monitoring Network was established in 1994 by the Taiwan Environmental Protection Agency (TEPA, now the Ministry of Environment) with a total of 76 stations. Stations were systematically sited based on regional pollutant emissions, meteorological conditions, and spatial concentration patterns (30). To ensure exposure assessments focused on densely populated areas, national park and background stations were excluded, leaving 71 active stations comprising general (*n* = 60), traffic (*n* = 6), and industrial (*n* = 5) sites to provide continuous data. Individual prenatal exposure was estimated by mapping the study population's residential postal codes across Taiwan's 368 townships to their respective nearest monitoring stations. Continuous, hourly data were converted into standardized regulatory metrics, representing monthly averages derived from 1-h sulfur dioxide (SO_2_), oxides of nitrogen (NO_X_), nitric oxide (NO) and nitrogen dioxide (NO_2_); 8-h carbon monoxide (CO), carbon dioxide (CO_2_), and ozone (O_3_) and 24-h particulate matter <10*μ*m (PM_10_) and particulate matter <2.5μm (PM_2.5_) measurements. The Pollutant Standards Index (PSI) is a composite metric that converts the concentrations of major air pollutants (such as PM_10_, SO_2_, NO_2_, CO, and O_3_) into a single standardized scale from 0 to 500 based on their potential health impacts. It is calculated using piecewise linear interpolation, where a sub-index is generated for each pollutant, and the highest sub-index on a given day becomes the overall PSI. Because the PSI is driven only by the single most severe pollutant, masking the concurrent levels of other pollutants, we handled it differently in our analysis. We treated the PSI as a broad, generalized indicator of overall daily air quality, whereas we analyzed the individual pollutant concentrations separately to evaluate the specific, independent epidemiological effects of each chemical exposure. Average prenatal exposure was quantified by calculating monthly mean pollutant concentrations linked to the maternal residential postal code at delivery, spanning the 40 weeks preceding birth.

### Regional analysis

Air pollution sources vary significantly across Taiwan due to distinct regional characteristics. In northern Taiwan, emissions are predominantly driven by traffic resulting from high population density and concentrated commercial activities. Conversely, southern Taiwan's air pollution is primarily industrial. Consequently, we conducted a regional analysis stratified by different counties and cities.

### Statistics

Statistical analyses were performed using SAS software, version 9.4 (SAS Institute, Cary, NC, USA). Quantitative data were expressed as frequencies and percentages for categorical variables, and as means with standard deviations for continuous variables. Continuous variables were compared using Student's *t*-test, whereas categorical variables were evaluated using Pearson's chi-square test. Multiple logistic regression models were constructed to assess the association between prenatal air pollutant exposure and the risk of TTN after adjusting for potential confounders. Adjusted odds ratios (aORs) and 95% confidence intervals (CIs) were calculated for each air pollutant. Statistical significance was defined as a two-sided *p*-value less than 0.05.

## Results

### Baseline characteristics of study population

A total of 15,455 newborns were enrolled in this study, comprising 3,091 cases in the TTN group and 12,364 matched controls. The baseline demographic and clinical characteristics of both groups are summarized in Table 1. Neonates in the TTN group exhibited significantly lower birth weights and shorter gestations compared to controls; specifically, 56.7% of the TTN group weighed less than 2,500 g (versus 7.3% in the control group), and 59.0% were born preterm at less than 37 weeks (versus 8.0% of controls). Cesarean delivery was significantly more prevalent among TTN cases than controls (63.5% vs. 36.9%). Additionally, mothers of infants with TTN experienced higher rates of comorbidities than those in the control group, including diabetes mellitus (2.3% vs. 0.8%), hypertension (3.5% vs. 1.0%), and pre-eclampsia (2.8% vs. 0.5%).

**Table 1:** The baseline demographic and clinical characteristics of both groups.

Characteristics	Control Group (*n* = 12,364)	TTN Group (*n* = 3,091)	*p*-value
	*n* (%)	*n* (%)	
Maternal Age (years)			
20–25	1,468 (11.9)	367 (11.9)	1.000
25–30	3,636 (29.4)	909 (29.4)	
30–35	4,332 (35.0)	1,083 (35.0)	
≥ 35	2,928 (23.7)	732 (23.7)	
Neonatal Gender			1.00
Female	5,312 (43.0)	1,328 (43.0)	
Male	7,052 (57.0)	1,763 (57.0)	
Birth weight (g)			< 0.001
≥ 2,500	11,457 (92.7)	1,338 (43.3)	
< 2,500	907 (7.3)	1,753 (56.7)	
Gestational age			< 0.001
≥ 37weeks	11,369 (92.0)	1,266 (41.0)	
< 37weeks	995 (8.0)	1,825 (59.0)	
Mode of delivery			< 0.001
Vaginal delivery	7,805 (63.1)	1,128 (36.5)	
Cesarean section	4,559 (36.9)	1,963 (63.5)	
Maternal comorbidity			
Asthma	518 (4.2)	149 (4.8)	0.12
Allergic rhinitis	3,211 (26.0)	791 (25.6)	0.67
Atopic dermatitis	460 (3.7)	106 (3.4)	0.44
Diabetes mellitus	95 (0.8)	70 (2.3)	< 0.001
Hypertension	125 (1.0)	108 (3.5)	< 0.001
Pregnancy-related complication			
Gestational hypertension	30 (0.2)	24 (0.8)	< 0.001
Gestational diabetes mellitus	192 (1.6)	58 (1.9)	0.20
Pre-eclampsia	60 (0.5)	86 (2.8)	< 0.001

### Air pollutant exposure and risk of transient tachypnea of the newborn

The associations between individual air pollutant exposures and the risk of TTN evaluated via multiple logistic regression models are presented in Table 2. After adjusting for potential confounders, including maternal age, neonatal sex, birth weight, delivery mode, preterm status, maternal comorbidities (asthma, allergic rhinitis, atopic dermatitis, diabetes mellitus, and hypertension), and pregnancy-related complications (gestational hypertension, gestational diabetes, and pre-eclampsia), multivariate analysis revealed that prenatal exposure to CO was significantly associated with an increased risk of TTN (aOR: 1.806, 95% CI: 1.312–2.487; *p* < 0.001). Significant associations with TTN risk were also observed for prenatal exposure to both NOx (aOR: 1.010, 95% CI: 1.005–1.015; *p* < 0.001) and PM_2.5_ (aOR: 1.001, 95% CI: 1.000–1.002; *p* = 0.023).

**Table 2:** Level of exposure to air pollutants during pregnancy and multivariable analysis for air pollutant and risk of transient tachypnea of the newborn (TTN)*.

Air pollutants	Level of exposure to air pollutants during pregnancy, mean (standard deviation)		aOR	95%CI	*p*-value
	Control Group (*n* = 12,364)	TTN Group (*n* = 3,091)			
SO_2_ (ppb)*10	39.52 (15.8)	39.56 (14.96)	1.029	0.997∼1.061	0.07
CO (ppm)*10	5.25 (1.49)	5.33 (1.61)	1.806	1.312∼2.487	< 0.001
O_3_ (ppb)*10	286.03 (45.92)	285.96 (48.22)	0.996	0.986∼1.007	0.47
PM_10_ (μg/m^3^)*10	546.5 (166.67)	537.61 (159.16)	1.002	0.999∼1.005	0.29
PM_2.5_ (μg/m^3^)*10	503.88 (690.24)	502.87 (686.77)	1.001	1.000∼1.002	0.023
NO_x_ (ppb)*10	249.8 (90.77)	254.38 (96.14)	1.010	1.005∼1.015	< 0.001
NO (ppb)*10	64.8 (34.64)	66.71 (36.51)	1.022	1.008∼1.036	0.002
NO_2_ (ppb)*10	194.08 (88.96)	196.2 (84.86)	1.006	1.001∼1.011	0.023

*Model adjusted for maternal age, neonatal gender, birth weight, mode of delivery, preterm delivery, maternal comorbidities (asthma, allergic rhinitis, atopic dermatitis, diabetes mellitus, hypertension), and pregnancy-related complications (gestational hypertension, gestational diabetes mellitus, and pre-eclampsia).

aOR: adjusted odds ratio, CI: confidence interval, SO_2_: sulfur dioxide, CO: carbon monoxide, O_3_: ozone, PM_10_: particulate matter <10μm, PM_2.5_: particulate matter <2.5μm, Nox: oxides of nitrogen, NO: nitric oxide, NO_2_: nitrogen dioxide.

### Regional variations in the impact of air pollutants

The regional analysis revealed that the association between prenatal carbon monoxide (CO) exposure and transient tachypnea of the newborn (TTN) risk was particularly pronounced in urban areas of northern Taiwan, including Taipei City (aOR: 3.473, 95% CI: 1.568 to 7.695; *p* = 0.002), New Taipei City (aOR: 13.832, 95% CI: 3.937 to 48.594; *p* < 0.001), and Taoyuan County (aOR: 11.885, 95% CI: 1.169 to 120.805; *p* = 0.036). In contrast, Kaohsiung City, a major industrial metropolitan area in southern Taiwan, exhibited an inverse association between prenatal CO exposure and TTN risk (aOR: 0.157, 95% CI: 0.042 to 0.579; *p* = 0.006) (Figure 2). Consistent patterns were observed for nitrogen oxides (NO_x_), which increased TTN risk in urban northern Taiwan (including Taipei City and New Taipei City) but showed an inverse association in Kaohsiung City (Figure 3). Additionally, prenatal exposure to PM_2.5_ demonstrated a statistically significant association with TTN risk exclusively in Taipei City (aOR: 1.003, 95% CI: 1.001 to 1.005; *p* = 0.023) (Supplementary Figure 1).

**Figure 2:**
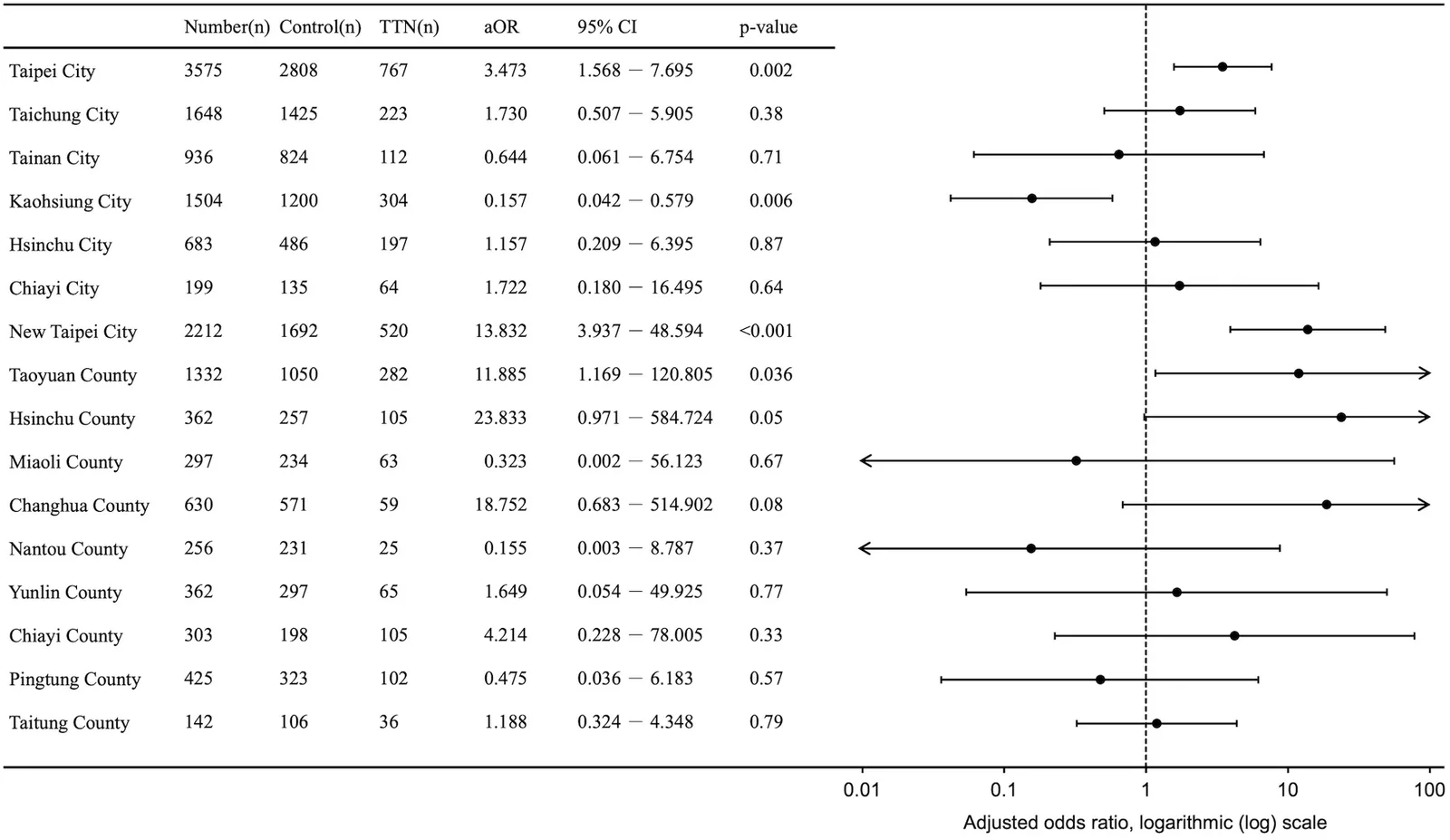
Regional analysis of the association between prenatal carbon monoxide (CO) exposure and the risk of transient tachypnea of the newborn (TTN). Data are presented as adjusted odds ratios (aORs) and 95% confidence intervals (CIs) across different counties and cities in Taiwan.

**Figure 3:**
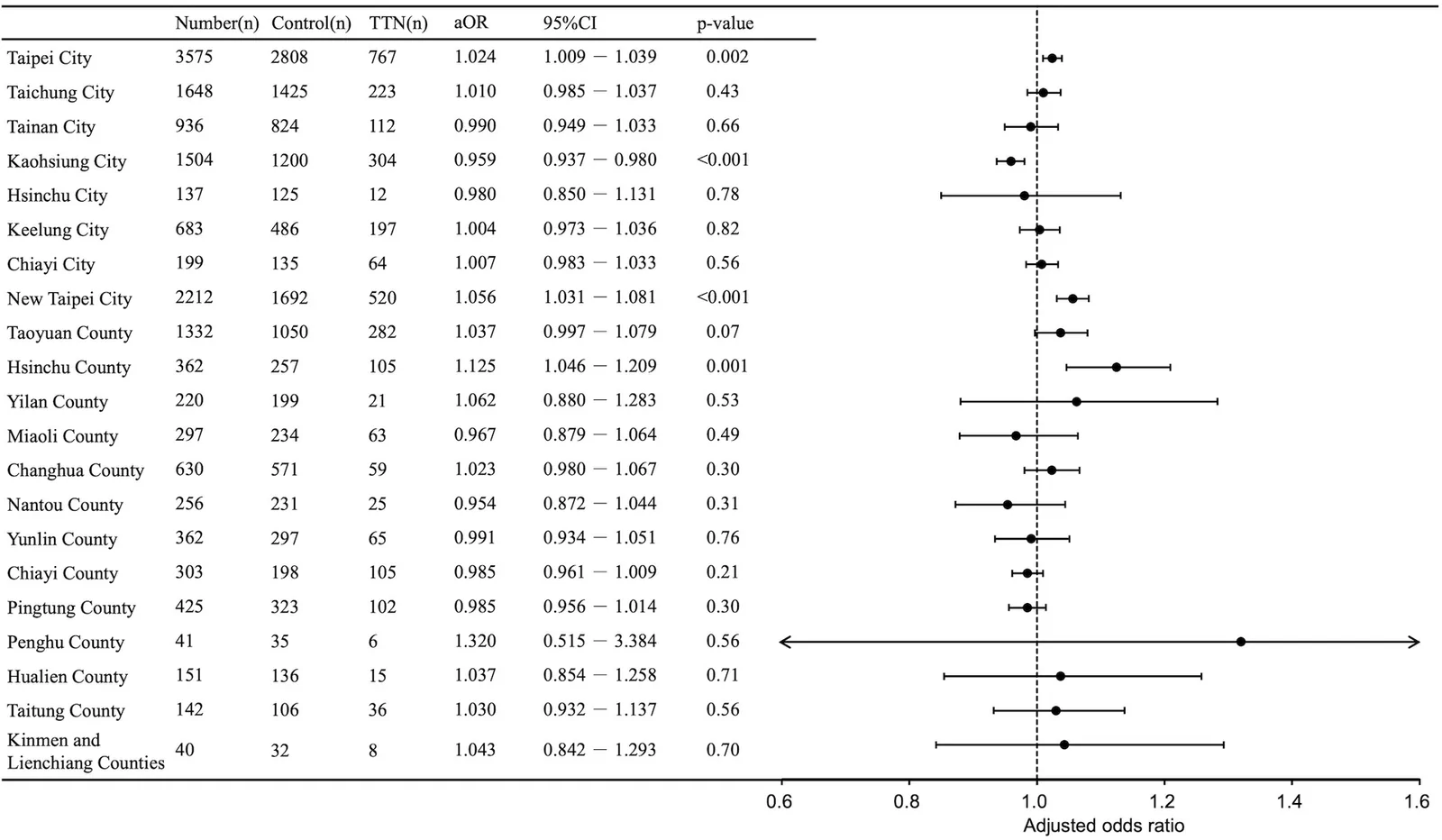
Regional analysis of the association between prenatal nitrogen oxides (no_x_) exposure and the risk of transient tachypnea of the newborn (TTN). Data are presented as adjusted odds ratios (aORs) and 95% confidence intervals (CIs) across different counties and cities in Taiwan.

### Trimester-specific analyses in the impact of air pollutants

Using multiple logistic regression, we analyzed associations between prenatal air pollution and TTN risk across trimesters (defined as ≤13, 14–27, and ≥28 gestational weeks).

CO exposure consistently increased TTN risk across the first (aOR: 1.735; 95% CI: 1.315–2.289; *p* < 0.001), second (aOR: 1.717; 95% CI: 1.289–2.285; *p* < 0.001), and third trimesters (aOR: 1.675; 95% CI: 1.249–2.247; *p* < 0.001). Similarly, nitrogen oxides (NOx, NO, NO₂) significantly elevated TTN risk throughout pregnancy. Although overall SO₂ exposure lacked significance (aOR: 1.029; 95% CI: 0.997–1.061; *p* = 0.072), positive associations emerged during the second (aOR: 1.032; 95% CI: 1.001–1.063; *p* = 0.045) and third trimesters (aOR: 1.039; 95% CI: 1.008–1.071; *p* = 0.013). Furthermore, while PM₂.₅ exposure was not significant during the second trimester, it remained significant for the overall pregnancy, as well as during the first (aOR: 1.003; 95% CI: 1.001–1.004; *p* < 0.001) and third trimesters (aOR: 1.003; 95% CI: 1.001–1.004; *p* = 0.003).

Overall, risk trends maintained a consistent direction across most pollutants and gestational periods. (Supplementary Figure 2).

## Discussion

This nationwide study demonstrated that prenatal exposure to specific air pollutants, including CO, NO_x_ and PM_2.5_ increased the risk of TTN. A major strength of our study is its large-scale, population-based design, which allowed for robust control of perinatal confounding factors. Moreover, our regional analysis demonstrated that varying sources of air pollution can lead to differential impacts across geographic areas.

Although the precise biological mechanisms linking prenatal air pollution to TTN remain incompletely understood, evidence suggests several plausible pathways. Maternal exposure to ambient pollution induces systemic inflammation, oxidative stress, and endothelial dysfunction within the maternal-placental unit, potentially disrupting fetal lung maturation. Particulate matter can cross the placental barrier, triggering direct fetal inflammatory cascades (31–33). In animal models, *in utero* PM_2.5_ exposure causes significant oxidative stress in fetal lung tissues, suppresses developmental genes, and impairs epithelial sodium channels critical for neonatal lung fluid clearance. This inflammation disrupts alveolarization and causes epithelial-mesenchymal transition, reducing fetal lung compliance (34). Furthermore, pollution-induced placental vascular dysregulation and functional impairment cause intrauterine hypoxia, indirectly hindering organogenesis (32, 35, 36). Epigenetic modifications also play a role, as prenatal pollution is associated with altered DNA methylation, histone modifications, and microRNA expression (34, 37–40). In utero exposure to ultrafine particles has even been shown to suppress early immune responses (41, 42). In line with these observations, a French cohort study suggests that prenatal PM_2.5_ exposure may adversely alter neonatal lung mechanics, pointing to reductions in functional residual capacity and tidal volume (43). Amid these varying institutional outcomes, our findings suggest a potential link between prenatal NO_x_ exposure and an increased risk of TTN.

Our regional analysis revealed distinct geographical variations, showing that the association between prenatal air pollution and TTN risk is more pronounced in urban Northern Taiwan. This region is a densely populated commercial metropolitan area where traffic emissions dominate (44, 45). These combustion-derived pollutants possess complex chemical compositions that may be highly biologically reactive at the maternal-fetal interface (46–50). Furthermore, the local basin topography limits atmospheric dispersion, trapping pollutants and causing sustained exposure (51). Conversely, Kaohsiung in Southern Taiwan exhibits an air pollution profile dominated by industrial, shipping, and secondary aerosol emissions (52). Its coastal location provides favorable meteorological conditions, including greater air circulation and less topographic confinement, which facilitate dispersion (53, 54). Together, these findings indicate that the relationship between air pollution and TTN may be influenced not only by concentration, but potentially also by pollutant sources, meteorological dynamics, and regional geographic factors. CO and NO_x_ are primary traffic-related emissions heavily concentrated in urban areas. In such environments, high levels of NO act as a scavenger for ozone (O3) via NO titration, depleting local O3 concentration. Because of this strong inverse spatial correlation in urban centers, the observed differences may be caused by varying combination effects with other pollutants, such as ozone (55). However, the inverse associations observed across regions should still be interpreted cautiously. The specific composition of regional pollutant mixtures may introduce synergistic or antagonistic health effects not fully captured by single-pollutant models. Additionally, residual confounding from unmeasured spatial variables, such as regional disparities in maternal lifestyle, socioeconomic status, or indoor air quality, cannot be completely ruled out. Finally, differences in sample sizes across these geographic areas may impact the statistical power and precision of the regional estimates, highlighting the need for further studies to validate these spatial disparities.

Several limitations in this study warrant consideration. First, reliance on ICD-9-CM and ICD-10-CM codes from claims data introduces a risk of diagnostic misclassification, though any such discrepancy should be balanced between groups. Second, although urbanization can affect healthcare accessibility, its confounding effect is likely limited due to Taiwan's small geographic size and the high accessibility afforded by the National Health Insurance (NHI) program. Third, assigning air quality monitoring stations by residential administrative district may not precisely reflect individual exposure, as it overlooks variations in lifestyle and personal mobility. Lastly, seasonal meteorological factors (including humidity, temperature, and wind patterns) were not incorporated into the current analysis. The ecological fallacy is difficult to avoid in conducting air pollution research, as we must assume that everyone within a specific region is equally exposed to the pollutants. We recognize that individual factors, such as working indoors versus outdoors, heavily influence actual exposure. However, privacy regulations prevented us from accessing detailed residential addresses for further analysis. Maternal body mass index and smoking status are potential contributing factors to TTN incidence. However, these variables are unavailable within NHIRD. While preconception air pollution exposure has been linked to adverse pregnancy outcomes (56, 57), the current study focused specifically on exposures during pregnancy. Further research is necessary to elucidate the epigenetic effects of air pollution during oogenesis and spermatogenesis. Regarding clinical significance and the seemingly small absolute differences, it is important to interpret the odds ratio in the context of continuous variables. In our model, the odds ratio for PM2.5 is calculated per specific unit increase (e.g., per 10 μg/m³ increment). While the average population differences between groups may appear narrow, an individual experiencing a 10 μg/m³ higher exposure faces a compounded risk exponentially related to this unit increment. Therefore, the findings demonstrate a clinically meaningful impact of pollution variations on TTN risk, even when the crude absolute mean differences across the entire cohort are small. The number of observations varied substantially across geographic strata. Larger metropolitan areas, including Taipei City and New Taipei City, contributed relatively larger numbers of participants and therefore yielded more precise estimates. In contrast, smaller geographic strata had limited sample sizes, as reflected by the wide confidence intervals observed for some estimates. Regarding the inconsistency between the crude PM₂.₅ exposure comparisons in Table 2 and the adjusted regression results, it is important to note that the data exhibit large standard deviations relative to their means. Consequently, direct crude comparisons cannot reliably indicate the true direction of the association, particularly because the underlying data are likely not normally distributed.

## Conclusion

This study demonstrated that prenatal exposure to specific air pollutants, including CO, NO_x_, and PM_2.5_, is associated with an increased risk of TTN. Furthermore, our regional analysis suggests that traffic-related emissions may exert the greatest impact. These findings underscore the importance of strengthening air quality monitoring and implementing targeted public health interventions.

## Data Availability

The data analyzed in this study is subject to the following licenses/restrictions: To protect patient confidentiality and ensure the reliability of the databases, investigators are required to conduct onsite analyses at the Health and Welfare Data Science Center in Taiwan via a remote connection to the Ministry of Health and Welfare servers. Requests to access these datasets should be directed to Dr. Ching-Heng Lin (epid@vghtc.gov.tw).

## Supplementary material

Supplementary Figure 1 Regional analysis of the association between prenatal particulate matter <2.5μm (PM2.5) exposure and the risk of transient tachypnea of the newborn (TTN). Data are presented as adjusted odds ratios (aORs) and 95% confidence intervals (CIs) across different counties and cities in Taiwan.

Supplementary Figure 2 Risk trends of air pollutants across different gestational periods.
